# The role of flumazenil in generalised anxiety disorder: a pilot naturalistic open-label study with a focus on treatment resistance

**DOI:** 10.1177/20451253231156400

**Published:** 2023-03-15

**Authors:** Alexander T Gallo, Stephen Addis, Vlad Martyn, Hishani Ramanathan, Grace K Wilkerson, Kellie S Bennett, Sean D Hood, Hans Stampfer, Gary K Hulse

**Affiliations:** Division of Psychiatry, Medical School, The University of Western Australia, Nedlands, WA, 6009, Australia; Fresh Start Recovery Programme, Subiaco, WA, Australia; Fresh Start Recovery Programme, Subiaco, WA, Australia; Division of Psychiatry, Medical School, The University of Western Australia, Nedlands, WA, Australia; Division of Psychiatry, Medical School, The University of Western Australia, Nedlands, WA, Australia; Division of Psychiatry, Medical School, The University of Western Australia, Nedlands, WA, Australia; Division of Psychiatry, Medical School, The University of Western Australia, Nedlands, WA, Australia; Division of Psychiatry, Medical School, The University of Western Australia, Nedlands, WA, Australia; Division of Psychiatry, Medical School, The University of Western Australia, Nedlands, WA, Australia; School of Medical and Health Sciences, Edith Cowan University, Joondalup, WA, Australia; Fresh Start Recovery Programme, Subiaco, WA, Australia

**Keywords:** DASS-21, flumazenil, anxiety, GABA, infusion, subcutaneous, treatment-resistant

## Abstract

**Background:**

Anxiety disorders are highly prevalent and chronic disorders with treatment resistance to current pharmacotherapies occurring in approximately one in three patients. It has been postulated that flumazenil (FMZ) is efficacious in the management of anxiety disorders via the removal of α_4_β2δ gamma-aminobutyric acid A receptors.

**Objective:**

To assess the safety and feasibility of continuous low-dose FMZ infusions for the management of generalised anxiety disorder (GAD) and collect preliminary efficacy data.

**Design:**

Uncontrolled, open-label pilot study.

**Method:**

Participants had a primary diagnosis of generalised anxiety disorder (GAD) and received two consecutive subcutaneous continuous low-dose FMZ infusions. Each infusion contained 16 mg of FMZ and was delivered over 96 ± 19.2 h. The total dose of FMZ delivered was 32 mg over approximately 8 days. Sodium valproate was given to participants at risk of seizure. The primary outcome was the change in stress and anxiety subscale scores on the Depression Anxiety Stress Scale–21 between baseline, day 8, and day 28.

**Results:**

Nine participants with a primary diagnosis of GAD were treated with subcutaneous continuous low-dose FMZ infusions; seven participants met the criteria for treatment resistance. There was a significant decrease in anxiety and stress between baseline and day 8 and baseline and day 28. There was also a significant improvement in subjective sleep quality from baseline to day 28 measured by the Jenkins Sleep Scale. No serious adverse events occurred.

**Conclusion:**

This study presents preliminary results for subcutaneous continuous low-dose FMZ’s effectiveness and safety in GAD. The findings suggest that it is a safe, well-tolerated, and feasible treatment option in this group of patients. Future randomised control trials are needed in this field to determine the efficacy of this treatment.

## Introduction

Anxiety disorders are a group of highly prevalent, chronic, and comorbid disorders that are ranked as the ninth most health-related cause of disability globally.^1^ The 12-month prevalence of generalised anxiety disorder (GAD) ranges from 0.2% to 4.3%,^2–4^ and lifetime prevalence ranges from 2.8% to 9.0%.^3–6^ Accordingly, GAD affects nearly one in ten people over a lifetime, and with most patients not in remission after 5–12 years,^7,8^ the disorder is complex and difficult to manage. GAD is further complicated by high comorbid rates of major depressive disorder (MDD), present in 52.6% of lifetime GAD cases, and any comorbid anxiety disorder occurring in 51.7% of lifetime GAD cases.^4^

While non-pharmacological interventions are the first-line management for GAD,^9–12^ depending on a number of factors,^10,13^ pharmacological interventions are often employed and typically involve treatment with selective serotonin reuptake inhibitors (SSRIs) or serotonin noradrenaline reuptake inhibitors (SNRIs).^11–14^ However, other pharmacological approaches have been used, including tricyclics, benzodiazepines (BZDs), pregabalin, quetiapine, buspirone, moclobemide, and more recently, agomelatine and vortioxetine.^14–19^

While SSRIs and SNRIs show efficacy in GAD, they are associated with side effects, including sexual dysfunction, nausea, and worsening of anxiety at the start of treatment, which can be bothersome for patients^20^ and may lead to discontinuation in as many as 22%.^21^ Compounding this, discontinuation of these drugs can result in a withdrawal syndrome, which is estimated to occur in 55.7% of patients.^22^ Symptoms of the withdrawal syndrome include anxiety, insomnia, irritability, shock-like sensations, dizziness, nausea, fatigue, and headaches.^23^ Given the commonality between the symptoms of SSRI/SNRI withdrawal and anxiety disorders, clinicians may incorrectly reinstate SSRI/SNRI treatment resulting in unnecessary continuation.^22,24^ In addition, the less commonly used treatments are associated with other limitations, such as abuse potential for BZDs and pregabalin^25^ and metabolic side effects for quetiapine.^26^ While there is an array of pharmaceuticals used in the management of GAD, each comes with its own limitations, including a significant number of patients not responding to existing pharmacotherapy and remaining treatment-resistant. As such, there is always a need to search for novel treatments that are efficacious, particularly for the estimated 30% of treatment-resistant patients.^27,28^

Dysfunction of the gamma-aminobutyric acid (GABA) system has been associated with anxiety disorders, and modulation of the GABA system can result in anxiolysis or anxiogenesis, whereby positive modulators of GABA type A (GABA_A_) receptors result in anxiolysis and negative modulators produce an anxiogenic effect.^29^ Typically, GABA_A_ receptors containing the α_2_ subunit (e.g. α_2_βγ_2_) are responsible for the anxiolytic effects of BZDs and are expressed in the hippocampus, cortex, striatum, and nucleus accumbens.^30^ Recently, it was theorised that flumazenil (FMZ), an antagonist at the allosteric BZD binding site on the GABA_A_ receptor,^31^ could be useful in the management of anxiety disorders (see Gallo and Hulse^32^ for review). The theory postulates that chronic stress results in paradoxical reactions to the endogenous neurosteroid allopregnanolone through alterations in the expression of certain GABA_A_ receptor subtypes and decreased GABA-mediated inhibition in the presence of allopregnanolone.^32–34^ FMZ has been shown to cause internalisation of these receptors, which may result in an anxiolysis independent of α_2_ subunit-containing GABA_A_ receptors.^32,35^ As chronic stress may be present in and related to GAD,^36^ theoretically FMZ may show efficacy in reducing GAD symptoms. However, administration of FMZ comes with several barriers: low bioavailability (16%), extensive first-pass metabolism, and short half-life (0.7–1.3 h).^37^ To overcome these barriers, FMZ has been delivered via a continuous infusion both intravenously and subcutaneously, primarily in the management of BZD withdrawal;^38^ however, this is the first study to investigate the theory of an anxiolytic action of FMZ in anxiety disorders, more specifically, in GAD. To test this theory, a small cohort of treatment- and non-treatment-resistant participants with a primary diagnosis of GAD received subcutaneous FMZ infusions. Treatment resistance was defined as having received or currently receiving a therapeutic dose of any pharmacotherapy for GAD for an adequate period (at least 6 weeks) and still experiencing clinically significant symptoms as assessed by the treating psychiatrist.

## Method

### Trial design

A small pilot naturalistic open-label observational study of participants being treated with subcutaneous FMZ infusions for GAD meeting the *Diagnostic and Statistical Manual of Mental Disorders–Fifth Edition* (*DSM*-5) criteria.

### Clinical setting

The study was conducted at an outpatient class B day hospital (Subiaco, Western Australia). The study was approved by Southcity Medical Centre Human Research Ethics Committee (001//2019) and recognised by the University of Western Australia Human Research Ethics Committee (2019/RA/4/20/5926). All participants gave written informed consent. Data were collected between March 2021 and June 2022.

### Participants

Participants were patients referred to the outpatient clinic for assessment and treatment for GAD symptoms using FMZ, with or without a history of treatment resistance. All participants underwent an assessment by the treating psychiatrist (S.A. or S.H.) and met the criteria in the *DSM*-5 for GAD.^39^ Inclusion criteria were: (1) met the *DSM*-5 diagnostic criteria for a primary diagnosis of GAD, (2) adult aged 18 years and above, and (3) willing and able to give informed consent for data collection. Exclusion criteria were: (1) had initiated or changed the dose of any psychotropic medication that could be used in the management of GAD (e.g. SSRIs and SNRIs) in the last 6 weeks, (2) had previously received low-dose continuous or implant FMZ for any indication, (3) currently pregnant or breastfeeding, (4) untreated hyperthyroidism, which may be a differential diagnosis for GAD, and (5) using BZDs daily as FMZ has been shown to reduce BZD use in high-dose users (⩾30 mg diazepam equivalents).^40^ The choice to exclude participants using BZDs daily was made to reduce the confounding effect that decreasing or ceasing BZDs may have on anxiety levels (i.e. increased anxiety from decreased GABAergic tone and/or precipitating withdrawal by decreasing BZD use). Participants taking BZDs on an as needed bases (i.e. not daily) were not excluded as FMZ is less likely to affect low-dose BZD users.^40^ In addition, it may have been difficult to find participants with treatment-resistant GAD who were not using any BZDs. Conversely, alcohol use was not an exclusion criterion as it has not been shown to be anxiolytic or anxiogenic in alcohol use disorders.^41^

### Intervention

Laboratory tests [full blood count (FBC), urea and electrolytes (U&E), liver function test (LFT), and thyroid function test (TFT)] were taken prior to the infusion as a routine procedure. TFT was measured only at baseline to exclude hyperthyroidism. Follow-up blood tests and FMZ blood levels were taken opportunistically as part of standard safety monitoring. FMZ blood levels were taken at least 6.5 h (five times the upper limit of the half-life) after the infusion start to allow for distribution and steady state to be achieved. The quantification of free FMZ was done by liquid chromatography–mass spectrometry/mass spectrometry (LC-MS/MS); the procedure is accredited by the National Association of Testing Authorities (accreditation number: 20224; site number: 24029; Go Medical Industries, Pty Ltd, Subiaco Western Australia).^42^

All participants received two consecutive subcutaneous continuous low-dose FMZ infusions inserted by nursing staff trained in the procedure. A subcutaneous butterfly needle was inserted into the anterior abdominal wall, lateral to the umbilicus, connected to flow control tubing (flow rate: 0.31 ml/h) and a syringe, which contained the FMZ solution (16 mg/30 ml/96± 19.2 h).^43^ The syringe was then inserted into the SpringFusor^®^ pump manufactured by Go Medical Industries Pty Ltd (Subiaco, Western Australia), which allowed participants to be ambulatory for the duration of the infusions. All participants needed to be released into the care of a nominated person for the first 24 h following the insertion of the FMZ infusion and were encouraged only do activities they felt comfortable completing while carrying the syringe. The subcutaneous route was chosen (instead of the intravenous route) as this procedure was shown to be comfortable in a cohort of 13 participants receiving FMZ for BZD withdrawal.^43^ Participants were told to return to the clinic to change the infusion syringe, tubing, and needle after 4 days; however, if this was not possible, participants were given a syringe to take home and change themselves, which they were instructed to store in the fridge until needed. Participants were trained at the appointment on how to change the syringe where necessary. Therefore, all participants received 32 mg of FMZ at an approximate rate of 4 mg/24 h for approximately 8 days.

The risk of seizures using low-dose FMZ in BZD withdrawal has been previously documented and sodium valproate has been used for seizure prophylaxis.^44^ As alcohol acts on the GABA_A_ receptor similarly to BZDs, sodium valproate 500 mg twice a day was given to participants with a history of alcohol misuse for seizure prophylaxis for the duration of the infusions and then ceased. To our knowledge, there are no known drug–drug interactions between FMZ and sodium valproate and it has been used as seizure prophylaxis in BZD withdrawal studies; however, interactions have not been explicitly investigated and may be possible due to the enzyme inhibition caused by sodium valproate.^45^ Notwithstanding, given the duration of sodium valproate treatment (i.e., 8 days), a clinically significant interaction is unlikely.

### Outcome measures

Participants completed questionnaires for the efficacy analysis at baseline and days 4, 8, 14, and 28 (±1 day). The primary efficacy outcome measure was the change in the Depression Anxiety Stress Scale–21 (DASS-21) score for the anxiety and stress subscales.^46^ The minimum clinically important difference (MCID) for the primary outcomes has been previously reported.^47^ The MCID for the stress and anxiety subscales were 3.18 and 4.04 based on a move from the inpatient to outpatient category described by Ronk *et al.*^47^ for the mean stress and anxiety values at baseline, day 8, and day 28. It is important to note that the values reported by Ronk *et al.*^47^ were multiplied by two to make scores comparable with the DASS-42. As such, the MCID values are half of those reported by Ronk *et al.*^47^

Secondary outcome measures included the DASS-21 score for depression,^46^ the six-item short form of the Spielberger State Anxiety Inventory (SSAI-6) score,^48^ and the Jenkins Sleep Scale (JSS).^49^ The JSS was only assessed at baseline and day 28 as the scale measures sleep-related issues over the past 30 days.

The DASS-21 is a validated and commonly utilised tool for assessing the negative emotional states of depression, anxiety, and stress.^50^ The DASS-21 has been validated in a three-factor structure, utilised by a diverse range of clinical and non-clinical, cultural, and ethnic groups.^51–54^ Higher scores indicate a higher frequency of experiencing negative emotional states.^46^ Of interest to this study, the stress subscale is most highly correlated with GAD.^55^

The SSAI-6 produces similar scores to the full 20-item Spielberger State Anxiety Inventory offering a briefer scale for participants and therefore, was chosen to reduce response errors and unanswered items.^48^

The JSS addresses four different sleep difficulties: initiating sleep, maintaining sleep, frequent waking across the night, and daytime sleepiness after normal sleep duration.^49^ The JSS was originally designed for clinical research; the scale has internal reliability and is validated in different patient cohorts.^49^

Adverse events were self-reported by participants meeting the inclusion criteria that commenced low-dose FMZ treatment.

### Statistical methods

Data were included for analysis if the participant had received at least one 16 mg FMZ infusion (approximately 4 days), provided a baseline, and met the inclusion criteria with no exclusions. Descriptive statistics were reported for all efficacy outcome measures. Differences between mean depression, anxiety, and stress scores (DASS-21) and SSAI-6 scores from baseline, day 8, and day 28 were measured using a repeated measures analysis of variance (ANOVA) where assumptions of normality, homogeneity of variance, and sphericity were met. The α value was set at 0.05. Pairwise comparisons were made with a Bonferroni-adjusted *p* value of 0.017 for DASS-21 and SSAI-6 outcomes. JSS scores were compared at baseline and day 28 using a paired-samples *t* test where assumptions of normality were met for scores and score differences.

One participant did not complete the SSAI-6 scale on day 28. As such, this missing value was imputed using the worst observation carried forward, which was the participant’s baseline value. Sensitivity analysis was completed using the best possible outcome for the SSAI-6, which is a score of 6.

## Results

### Participant flow and characteristics

**Figure 1.**
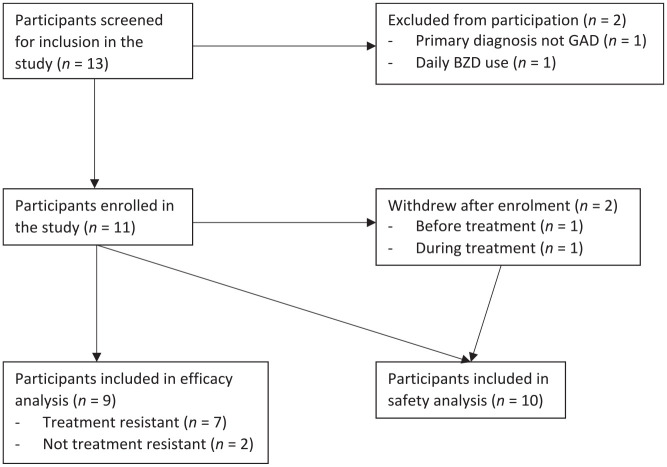
Flow chart of participant enrolment in the study. The one participant who withdrew during treatment was included in the safety analysis but not the efficacy analysis.

Eleven participants met the inclusion criteria and were recruited. Two participants were excluded from the efficacy analysis due to withdrawal from treatment. One participant withdrew before treatment commenced and the other withdrew during the first infusion due to a seized syringe with an estimated dose of 13 mg of FMZ delivered (42% of total dose). As such, this participant did not receive the anticipated therapeutic dose of FMZ and was excluded from the efficacy analysis; however, their safety outcomes were still included (Figure 1).

Nine participants were included in the efficacy analysis. The sample comprised five males and four females (Table 1). The mean age was 39.6 years ranging from 22 to 64 years. Most patients had a comorbid psychiatric condition and seven participants had trialled at least one pharmacotherapy for anxiety for an adequate period at a therapeutic dose and still experienced symptoms. Five of these participants were receiving pharmacological treatment at baseline for anxiety and were still experiencing anxiety symptoms. They were maintained on their medication during the FMZ infusion and the follow-up period. Participants taking BZDs or hypnotics were using them on an as needed basis and not daily; however, no participants reported BZD use during the infusion period. No participants had a personality disorder, received FMZ previously, or a history of seizures.

**Table 1.** Participant characteristics at baseline.

Male/female	5 (56)/4 (44)
Age, years (SD)	39.6 (15.6)
Height, cm (SD)	166.1 (12.9)^a^
Weight, kg (SD)	83.1 (43.2)^b^
History of anxiety disorder, years (SD)	12.1 (5.6)
Treatment resistant	7 (78)
Receiving psychotherapy	3 (33)
Relationship	
De facto/partner	1 (11)
Married	4 (44)
Separated	1 (11)
Single	3 (33)
Employment	
Full-time	3 (33)
Homemaker	2 (22)
Part-time/casual	2 (22)
Student	1 (11)
Unemployed	1 (11)
Education	
Secondary school	3 (33)
College/TAFE	1 (11)
Primary school	1 (11)
Tech/trade	1 (11)
Undergraduate	2 (22)
Postgraduate	1 (11)
Accommodation	
House or flat	9 (100)
Living	
Child(ren) and partner/spouse	2 (22)
Alone	1 (11)
Spouse/partner	2 (22)
With child(ren) only	1 (11)
Parent(s)	3 (33)
Co-morbid psychiatric conditions	6 (67)
Alcohol use disorder	2 (22)
Major depressive disorder	1 (11)
Post-traumatic stress disorder	1 (11)
Social anxiety	2 (22)
Taking psychoactive medication	5 (56)
SSRI	2 (22)
SNRI	1 (11)
Unclassified antidepressant^c^	2 (22)
Stimulant (e.g., dexamfetamine)	1 (11)
BZD/hypnotic	2 (22)
Naltrexone	1 (11)
Sodium valproate for seizure prophylaxis	2 (22)

BZD, benzodiazepines; SD, standard deviation; SNRI, serotonin noradrenaline reuptake inhibitor; SSRI, selective serotonin reuptake inhibitor; TAFE, Technical and Further Education.

Results reported as count (%) unless otherwise specified.

a Data missing for two participants.

b Data missing for one participant.

c Unclassified antidepressants included agomelatine and bupropion.

### Stress and anxiety

**Figure 2.**
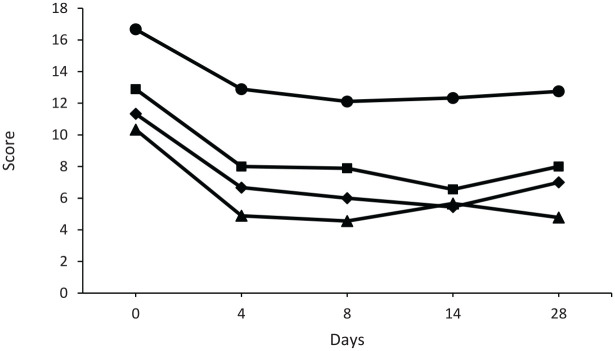
Depression, anxiety, and stress scores from the DASS-21 and state anxiety scores from the SSAI-6. Depression (◆), anxiety (▲), and stress (■) scores from the DASS-21 and state anxiety (•) scores from the SSAI-6. Day 0 denotes baseline.

The ANOVA results showed that stress scores on the DASS-21 (*n* = 9) varied significantly across the three timepoints, *F* (2, 16) = 11.08, *p* < 0.001, partial η^2^ = 0.58. Pairwise comparisons further revealed that stress levels at day 8 (M = 7.89, SD = 5.16, *p* = 0.003) and day 28 (M = 8.00, SD = 4.42, *p* = 0.012) were significantly lower than baseline (M = 12.89, SD = 5.04). Mean stress scores on days 4 and 14 were 8.00 (SD = 5.03) and 6.56 (SD = 4.50), respectively (Figure 2). The reduction from baseline to day 8 and baseline to day 28 exceeded the MCID and was clinically important.

The ANOVA results showed that anxiety scores on the DASS-21 (*n* = 9) varied significantly across the three timepoints, *F*(2, 16) = 15.06, *p* < 0.001, partial η^2^ = 0.65. Pairwise comparisons further revealed that anxiety levels at day 8 (M = 4.56, SD = 3.87, *p* < 0.001) and day 28 (M = 4.78, SD = 3.70, *p* = 0.013) were significantly lower than baseline (M = 10.33, SD = 3.46). Mean anxiety scores on days 4 and 14 were 4.89 (SD = 3.59) and 5.67 (SD = 4.03), respectively (Figure 2). The reduction from baseline to day 8 and baseline to day 28 exceeded the MCID and was clinically important.

The ANOVA showed SSAI-6 scores (*n* = 9) did not vary significantly across baseline (M = 16.67, SD = 4.56), day 8 (M = 12.11, SD = 4.65), and day 28 (M = 12.78, SD = 4.97), *F* (2, 16) = 2.88, *p* = 0.086. Sensitivity analysis using the best outcome score did not change the statistical significance of this outcome. Mean SSAI-6 scores on days 4 and 14 were 12.89 (SD = 4.14) and 12.33 (SD = 5.77), respectively.

### Depression

The mean baseline depression score was 11.33 (SD = 4.24) and decreased to 6.67 (SD = 5.36) on day 4, 6.00 (SD = 3.91) on day 8, 5.44 (SD = 4.42) on day 14, and slightly increased to 7.00 (SD = 6.95) on day 28 (Figure 2). The ANOVA showed depression scores (*n* = 9) varied significantly across the three timepoints (baseline, day 8, and day 28), *F* (2, 16) = 4.65, *p* = 0.026, partial η^2^ = 0.37. However, pairwise comparisons did not reveal any significant differences between any of the timepoints (*p* > 0.05).

### Sleep

A paired-samples *t* test was used to compare mean JSS scores (*n* = 9) between baseline (M = 11.89, SD = 3.48) and day 28 (M = 8.11, SD = 3.62). On average, the participant’s scores were 3.78 points lower [95% confidence interval (CI) = 0.45–7.10] after treatment with FMZ. This difference was statistically significant, *t*(8) = 2.62, *p* = 0.031, Hedges’ *g* = 0.79.

### FMZ blood levels

**Figure 3.**
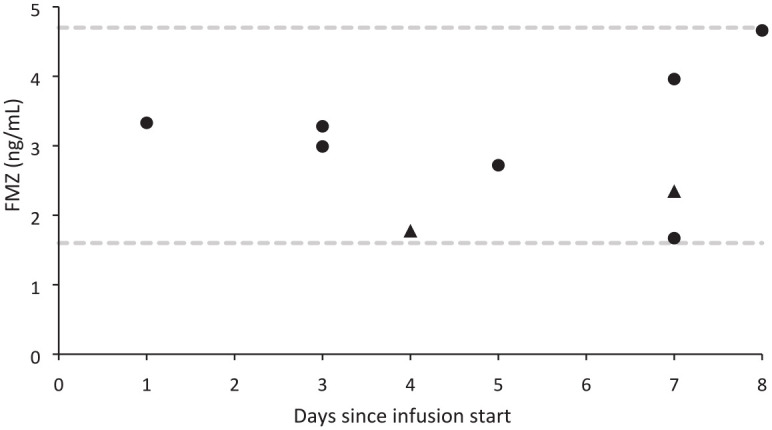
FMZ blood levels collected from participants on days 1–8 from the beginning of the infusion. Results are from eight participants. Day 0 denotes baseline. One participant provided two blood levels on days 4 and 7, which are represented with ▲. The maximum level was observed on day 8 at 4.66 ng/ml; the minimum level was observed on day 7 at 1.67 ng/ml.

Eight participants provided FMZ blood levels during the infusion (Figure 3). One participant provided two samples, making nine available blood FMZ samples. The maximum level observed was on day 8 (4.66 ng/ml) of the infusions and the lowest level was observed on day 7 (1.67 ng/ml) of the infusions.

### Adverse events

Overall, 14 adverse events were reported by eight participants during the infusion period (Table 2). The most common was fatigue, occurring in 50% of participants followed by itchiness or rash around the infusion site, which was likely due to the adhesive tape used to keep the needle in place. It is unclear whether the transient discolouration of urine was due to FMZ. No participants experienced a seizure, any serious adverse event, or reported any type of withdrawal syndrome. There were no remarkable changes in routine laboratory findings (FBC, U&E, and LFT) for participants that had a follow-up blood test within 28 days of starting FMZ treatment (*n* = 5).

**Table 2.** Participants’ self-reported adverse events.

Adverse events during FMZ infusion	Number of participants experiencing event
Fatigue	5 (50)
Stinging at injection site	1 (10)
Itchiness/rash around infusion site	3 (30)
Nausea	1 (10)
Bruising, swelling, or oedema around injection site	2 (20)
Heightened anxiety (transient)	1 (10)
Bright yellow urine^a^	1 (10)

FMZ, flumazenil.

Results reported as count (%) based on 10 participants.

a It is unclear whether this was related to FMZ.

## Discussion

Despite a small cohort of participants, a significant and clinically important reduction in anxiety and stress levels, measured using the DASS-21, and significant improvements in subjective sleep quality, measured using the JSS, were observed. While the inherent limitations of an open-label, uncontrolled study prevent the synthesis of any conclusions about the efficacy of treatment, this pilot study provides a feasible study design to evaluate the efficacy of treatment if applied in a randomised and controlled setting. Of high importance in future study designs is the significant difference in the stress subscale from the DASS-21, as this is most effective at evaluating symptoms corresponding to GAD.^55^ In addition, neither the DASS-21 nor the SSAI-6 measures sleep disturbances, which is a symptom listed in the *DSM*-5 for GAD and, therefore, the improvement in the JSS score is consistent with an improvement in GAD symptoms.^39^

Treatment-resistant anxiety disorder patients have been shown to have a very poor quality of life and a high rate of suicide attempts.^28^ Accordingly, anxiety disorders have a serious impact on health, both mental and physical, and represent a significant cost burden to healthcare systems. This is explained by multiple medical evaluations and the treatment of physical manifestations (e.g., muscle pains, aches, and chest pain) coupled with a decreased quality of life and productivity.^28,56^ This highlights the pertinence of further evaluating pharmacological options for the treatment of these resistant disorders, while minimising the common side effects associated with other commonly prescribed drugs to reduce these impacts. Importantly, in this cohort of participants, there were no reports of a withdrawal syndrome, and the troublesome side effect of sexual dysfunction was also not observed, which is commonly seen with SSRIs and SNRIs.^22^ The most commonly experienced adverse event was fatigue, which may be indicative of increased GABAergic tone. Although FMZ is typically an antagonist at the BZD binding site of the GABA_A_ receptor, there are data that demonstrate FMZ acts as a positive allosteric modulator at α_4_ containing GABA_A_ receptors, which may account for the fatigue experienced during the infusions.^57–59^ Alternatively, fatigue is a symptom of MDD and may represent a symptom of this disorder; however, only one patient had this diagnosis at baseline.

DASS-21 was used to monitor changes in depression symptoms as depression is highly comorbid with anxiety disorders.^4^ While the ANOVA was significant, pairwise comparisons with a Bonferroni adjustment did not reveal any differences between the mean depression scores from baseline to days 8 and 28. The changes in the DASS-21 depression score may be explained by the high degree of overlap between the *DSM*-5 diagnostic criteria for MDD and GAD.^60^

FMZ’s efficacy in the management of anxiety disorders has been postulated to be related to the release of the neurosteroid, allopregnanolone, which increases in response to acute stress^61^ and decreases in response to chronic stress.^62–65^ Consequently, the GABA_A_ receptor subunit conformation has been demonstrated to change after chronic exposure to and withdrawal from allopregnanolone. This results in increased expression of α_4_β2δ GABA_A_ receptors, which are less sensitive to GABA-induced hyperpolarisation and may contribute to anxiety symptoms due to decreased inhibition.^32^ Since FMZ has been shown to decrease cell surface expression of α_4_β2δ GABA_A_ receptors,^35^ it was hypothesised that treatment with FMZ could result in anxiolytic effects that last beyond the duration of treatment.^32^ Results from this study support this theory; however, future randomised control studies are needed to determine the efficacy of FMZ infusions in the management of GAD.

## Limitations and strengths

The main limitations of this study are the small sample size and the open-label design limiting the interpretation of FMZ’s effect; however, as a pilot study, it has provided the information required to assess the feasibility of future clinical trials. The participants represented in this small cohort had several comorbid psychiatric conditions. While this could be seen as a limitation as a specific treatment population is not defined, this is also a strength as it provides data on the use of FMZ in a more common presentation of GAD, which will often involve comorbid psychiatric disorders. The efficacy, safety, and tolerability profile cannot be generalised until randomised control trials with large sample sizes of GAD participants are conducted. Finally, the use of sodium valproate may have confounded the anxiety scores at day 8 due to its mood stabilising effects; however, it is important to highlight that only two participants received sodium valproate up to day 8, and results at day 28 were still significant. Nevertheless, despite the small sample size, there was still a significant difference in anxiety measures from baseline in a predominately difficult to treat population with treatment-resistant anxiety. These changes also occurred in participants who were currently receiving pharmacotherapy. No participants needed to discontinue their current pharmacotherapy to receive FMZ, which prevented confounding the results with potential withdrawal syndromes.

## Further research

While these results indicate that FMZ may be efficacious in the management of GAD, randomised control trials are required to make a conclusion on the efficacy of treatment. Although the subcutaneous route of administration has been favoured in the literature more recently,^38^ differences between the intravenous route should be explored (e.g. bioavailability and *C*_max_), particularly in BZD users where bolus doses have been shown to precipitate withdrawal,^66–71^ suggesting an anxiogenic effect of FMZ at certain concentrations. While the infusion procedure is more invasive than current oral first-line treatment options, such as SSRIs, any active comparator (pharmacotherapy) study designs should also assess the acceptability of this procedure and the side effects compared with standard oral daily treatments. Since seizures have been reported in trials assessing FMZ for BZD withdrawal,^44^ a larger cohort of patients that would not have pharmacodynamic GABA_A_ receptor changes from chronic BZD use needs to be assessed. In addition, as treatment-resistant GAD patients are often treated with BZDs, FMZ should also be investigated in this cohort to determine whether BZD use can be decreased or ceased while still observing an improvement in anxiety levels. While most participants did not receive sodium valproate for seizure prophylaxis, the precipitation of a seizure from FMZ cannot be ruled out in non-BZD using patients, which should be considered when designing larger clinical trials.

## Conclusion

Significant reductions in anxiety symptoms in participants with a primary diagnosis of GAD, most of whom were treatment-resistant to one or more pharmacotherapies, were observed on the anxiety and stress subscales of the DASS-21 in an open-label uncontrolled study design. These pilot data suggest that FMZ is safe in the management of GAD with or without treatment resistance and, as such, further research should be directed to confirm these results and determine the efficacy in a randomised and controlled setting.

## Declarations

**Ethics approval and consent to participate:** The study was approved by Southcity Medical Centre Human Research Ethics Committee (001//2019) and recognised by the University of Western Australia Human Research Ethics Committee (2019/RA/4/20/5926). All participants gave informed consent for participation in this study.

**Consent for publication:** All participants gave written informed consent for publication of their unidentifiable data.

**Availability of data and materials:** Data are not available as ethics approval for the sharing of data was not sought.
